# Free radicals properties of gamma-irradiated penicillin-derived antibiotics: piperacillin, ampicillin, and crystalline penicillin

**DOI:** 10.1007/s00411-013-0498-1

**Published:** 2013-11-09

**Authors:** Sławomir Wilczyński, Barbara Pilawa, Robert Koprowski, Zygmunt Wróbel, Marta Ptaszkiewicz, Jan Swakoń, Paweł Olko

**Affiliations:** 1 Department of Biophysics, School of Pharmacy and Laboratory Medicine, Medical University of Silesia in Katowice, Jedności 8, 41-200 Sosnowiec, Poland; 2 Faculty of Computer Science and Material Science, Institute of Computer Science, University of Silesia, Będzińska 39, 41-200 Sosnowiec, Poland; 3 Department of Radiation Physics and Dosimetry, Institute of Nuclear Physics, Polish Academy of Sciences, Radzikowskiego 152, 31-342 Cracov, Poland

**Keywords:** Radiosterilization, Paramagnetic centers, Free radicals, EPR spectroscopy, Antibiotics, β-Lactam antibiotics

## Abstract

The aim of this work was to determine the concentrations and properties of free radicals in piperacillin, ampicillin, and crystalline penicillin after gamma irradiation. The radicals were studied by electron paramagnetic resonance (EPR) spectroscopy using an X-band spectrometer (9.3 GHz). Gamma irradiation was performed at a dose of 25 kGy. One- and two-exponential functions were fitted to the experimental data, in order to assess the influence of the antibiotics’ storage time on the measured EPR lines. After gamma irradiation, complex EPR lines were recorded confirming the presence of a large number of free radicals formed during the irradiation. For all tested antibiotics, concentrations of free radicals and parameters of EPR spectra changed with storage time. The results obtained demonstrate that concentration of free radicals and other spectroscopic parameters can be used to select the optimal parameters of radiation sterilization of β-lactam antibiotics. The most important parameters are the constants *τ* (*τ*_1(*A*),(*I*)_ and *τ*_2(*A*),(*I*)_) and *K* (*K*_0(*A*),(*I*)_, *K*_1(*A*),(*I*)_, *K*_2(*A*),(*I*)_) of the exponential functions that describe free radicals decay during samples storage.

## Introduction

In modern medicine, a number of sterilization methods are applied, including tempering, cauterization, hot air sterilization, steam sterilization, sterile filtration, radiation sterilization (e.g., by ionizing radiation or UV light), gas sterilization (e.g., by ethylene oxide or formaldehyde), and chemical sterilization (Basly et al. 1996, 1997, 1998a, b, c; Varshney and Dodke 2004; Katušin-Ražem et al. 2005; Gibella et al. 2000; Fauconnet et al. 1996; Jacobs 1985).

Sterilization is intended to kill or remove all vegetative and sporing microbes from the environment or material (Wilczyński 2009). In the case of medical substances, choice of the sterilization method depends on the type, properties, and production method of the substance in question. Irradiation of drugs, or other medical products, by a suitable dose of ionizing radiation, conducted in an appropriate environment, ensures sterile conditions (Basly et al. 1996, 1997, 1998a, b). Basic terms and sterilization protocols can be found in PN-EN 552 (1999), PN-EN 556 (2005), and ISO 11137-1 (2006). Radiation sterilization is especially useful in the case of thermolabile products, because irradiation causes only a small rise in the temperature of sterilized substances (Basly et al. 1998b). In the case of gamma radiation, a substance to be sterilized does not directly interact with the reagents and, as a result, lacks any traces of chemical pollution (Katušin-Ražem et al. 2005). Moreover, packaged products may also be irradiated as gamma radiation possesses excellent penetrative properties; this constitutes one of its economic advantages (Basly et al. 1998a; Varshney and Dodke 2004; Katušin-Ražem et al. 2005). Note that medical products subjected to gamma radiation sterilization do not become radioactive (Katušin-Ražem et al. 2005).

## Materials and methods

In the present study, the penicillin-derived antibiotics, such as piperacillin, ampicillin, and crystalline penicillin, were used. All investigated antibiotics were supplied by Sigma-Aldrich. During the stability tests of free radicals, all samples were stored at room temperature in air, at 45–65 % relative humidity in the dark.

The samples were irradiated at room temperature in air by gamma radiation using a THERATRON 780E cobalt source containing ^60^Co with an activity of 128.4 TBq. Three samples of each antibiotic were irradiated.

In accordance with (PN-EN 552 1999), every tested antibiotic was subjected to a radiation dose equal to 25 kGy. All samples were located at optimal distance from the radiation source so that the dose gradient did not affect the homogeneity of the dose absorbed by the sample volume. Irradiation time was 155 min. Electron paramagnetic resonance (EPR) measurements were taken by means of an EPR spectrometer—SE/X (Radiopan)—with 100 kHz magnetic field modulation; X-band microwave radiation with a frequency of 9.3 GHz was used. The frequency of the microwave radiation was measured by MCM 101 (EPRAD). EPR spectra were recorded in the form of the first-derivative absorption by 2-mW microwave radiation, where microwave saturation cannot be observed. The radiation source of the EPR spectrometer was a klystron that supplied a power of 70 mW. The following EPR parameters were recorded: amplitude A (±0.05 a.u.), integral intensity I (±0.05 a.u.), line width Δ*H*_pp_ [mT] (±0.02 mT), and g-factor (±0.0002). Maximal errors of the analyzed parameters are given in brackets.

The values of amplitude and integral intensity of the EPR lines correspond to the number of paramagnetic centers in the sample. The amplitude was determined by the identification of the peak-to-peak of the first derivative of the EPR lines, while the integral intensity was determined by double integration of the EPR lines, which corresponds to the area under the curve of the absorption of microwaves.

The concentration of free radicals *N* in the tested antibiotics was calculated using the following formula:

$$N = n_{\text{U}} \cdot \frac{{I_{\text{P}} }}{{I_{\text{U}} }} \cdot \frac{{W_{\text{U}} \cdot A_{\text{Ru}} }}{{W_{\text{P}} \cdot A_{\text{Rp}} \cdot m_{\text{P}} }},$$

where *n*_U_ represents the number of paramagnetic centers in the ultramarine standard (1.18 × 10^19^ spin/g), *I*_P_ and *I*_U_ represent integral intensities of the EPR lines in the tested sample and the ultramarine standard, *A*_Rp_ and *A*_Ru_ represent amplitude of ruby EPR lines for the sample and ultramarine, *W*_P_ and *W*_U_ represent amplification factors for the sample and ultramarine standard, and *m*_P_ represents the sample mass. The maximal error of free radicals concentration *N* is ±0.1 × 10^17^ spin/g.

As the area under the absorption curve is proportional to the number of paramagnetic centers, the results of double integration of the first-derivative EPR signal were compared with those of the standard, and the number of paramagnetic centers per gram was determined for every sample.

Ultramarine (sodium aluminosilicate—g-factor 2.0290) was used as a reference for the concentrations of paramagnetic centers, while ruby crystal was used as an additional reference sample placed in the resonance cavity. Note that ultramarine is a standard with a stable spin concentration (*c* = 1.18 × 10^19^ spin/g), resistant against air and higher temperatures, and also a convenient standard of the resonance magnetic field having an isotropic g-factor of 2.0290 ± 0001 and a nearly ideal Lorentzian line shape in the middle part of the EPR spectrum. The wings of the ultramarine spectrum are Gaussian in shape.

Analysis of EPR integral intensities and amplitudes of the tested medicinal substances was performed as a function of storage time (*t*), by means of exponential functions fitted to the data, in an effort to quantify any changes in free radicals’ concentration in the sample. All samples were stored in thin-walled, open quartz tubes.

One- and two-exponential functions were used to analyze time patterns of EPR parameters:

1a $$I(t) = K_{0(I)} + K_{1(I)} \cdot \exp \left( {\frac{ - t}{{\tau_{1(I)} }}} \right)$$

1b $$I(t) = K_{0(I)} + K_{1(I)} \cdot \exp \left( {\frac{ - t}{{\tau_{1(I)} }}} \right) + K_{2(I)} \cdot \exp \left( {\frac{ - t}{{\tau_{2(I)} }}} \right)$$

2a $$A(t) = K_{0(A)} + K_{1(A)} \cdot \exp \left( {\frac{ - t}{{\tau_{1(A)} }}} \right)$$

2b $$A(t) = K_{0(A)} + K_{1(A)} \cdot \exp \left( {\frac{ - t}{{\tau_{1(A)} }}} \right) + K_{2(A)} \cdot \exp \left( {\frac{ - t}{{\tau_{2(A)} }}} \right)$$

Where *K*_0(*A*),(*I*)_ represents a constant, *K*_1(*A*),(*I*)_ and *K*_2(*A*),(*I*)_ represent the amplitudes of the exponential functions, and *τ*_1(*A*),(*I*)_ and *τ*_2(*A*),(*I*)_ are time constants.

Identification of the parameters *K*_0(*A*),(*I*)_, *K*_1(*A*),(*I*)_, *K*_2(*A*),(*I*)_, *τ*_1(*A*),(*I*)_, and *τ*_2(*A*),(*I*)_ was performed by Gauss–Newton and Levenberg–Marguardt methods (Björck and Dahlquist 1987) for the space of analyzed parameters, with a randomly set range. The best fit was obtained by minimizing the deviation of δ error between measured and simulated integral intensities and amplitudes as calculated below.${{\updelta}} = \frac{ 1}{\text{N}}\sum\limits_{n = 1}^{N} {\left| {A_{n} - } \right.\left. {A_{n}^{(S)} } \right|}$ ${{\updelta}} = \frac{ 1}{\text{N}}\sum\limits_{n = 1}^{N} {\left| {I_{n} - } \right.\left. {I_{n}^{(S)} } \right|} ,$where *A*_*n*_ and *A*_*n*_^S^ represent experimental and simulated amplitudes, *I*_*n*_ and *I*_*n*_^S^ represent experimental and simulated integral intensities, and N represents the number of measurements.

A characteristic feature of the adopted error criterion is to reduce the impact of large individual differences—outliers analysis (so-called Manhattan distance). In contrast, if the usual least squares procedure is applied where the differences are squared, a single large difference would significantly affect the result.

In order to perform a sensitivity analysis, the parameters *K*_0(*A*),(*I*)_, *K*_1(*A*),(*I*)_, *K*_2(*A*),(*I*)_, *τ*_1(*A*),(*I*)_ and *τ*_2(*A*),(*I*)_ were varied and the subsequent influence of these variations on the δ error was calculated using the following formula:$\delta_{{}}^{{}}$<$\hbox{min} (\delta )$·*p*_r_where *p*_r_ is a constant between 1.05 and 1.10.

This formula defines the parameter space in 2D or 3D systems for which the error does not increase by 5 % (*p*_r_ = 1.05) or 10 % (*p*_r_ = 1.10). Adjustment of model parameters in line with mathematical function was made by MATLAB (Koprowski and Wróbel 2008).

**Fig. 1:**
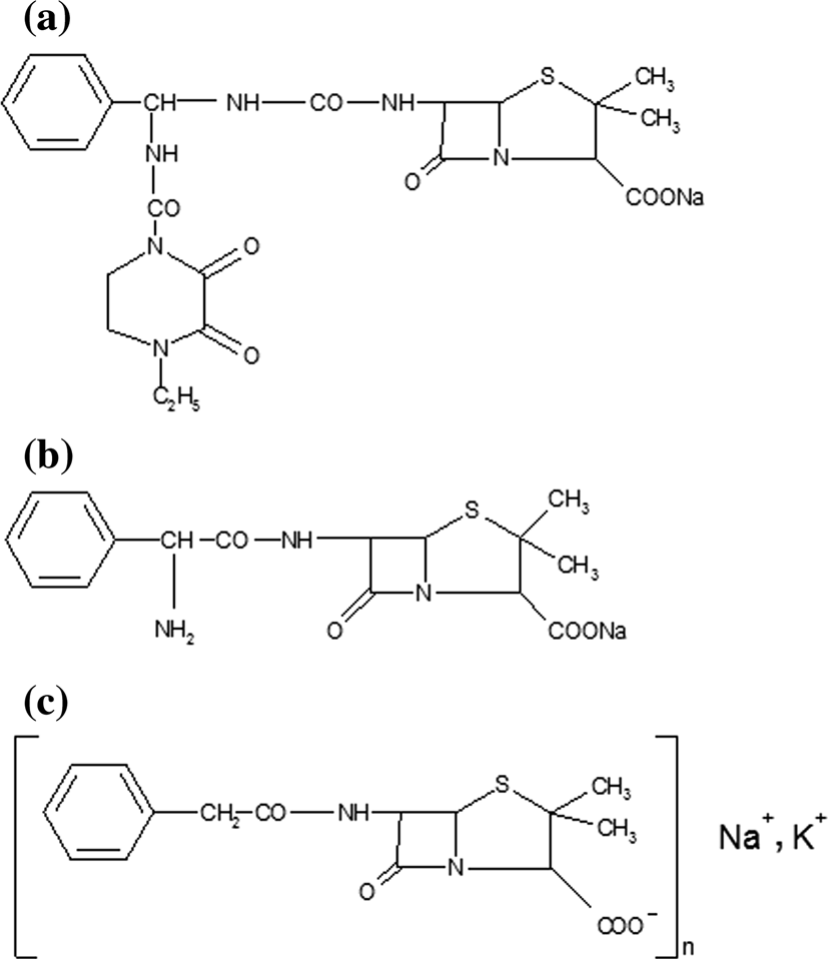
Chemical structure of the studied antibiotics: piperacillin (**a**), ampicillin (**b**), and crystalline penicillin (**c**) (Zejc and Gorczyca 2005)

The following penicillin-derived antibiotics, belonging to the group of β-lactam antibiotics (Fig. 1), were chosen for the study: piperacillin, ampicillin, and crystalline penicillin. Penicillin exerts its therapeutic effect by inhibiting the synthesis of bacterial walls. Because it is effective only against those cells that synthesize peptidoglycan, it is not toxic to humans (Janiec 2001). Four-member β-lactam ring fused to the five-member thiazolidine ring forms the fundamental structural components of penicillin-derived medications (Janiec 2001; Dzierżanowska 2000; Kostowski and Herman 2003).

## Results and discussion

Identification and dosimetry of paramagnetic centers in gamma-irradiated penicillin-derived antibiotics.

**Fig. 2:**
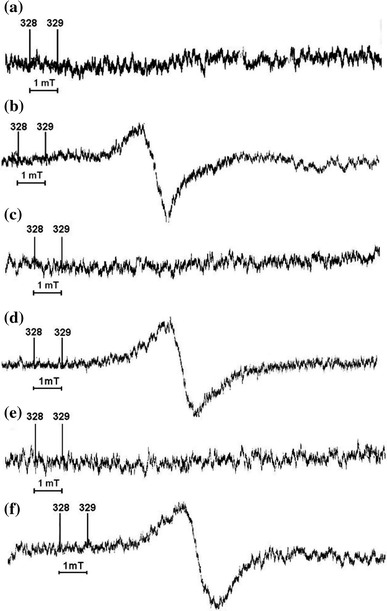
EPR spectra of the unirradiated and irradiated penicillin-derived antibiotics piperacillin (**a**, **b**), ampicillin (**c**, **d**), and crystalline penicillin (**e**, **f**). Microwave power—2 mW. EPR spectra were recorded on the same day when the gamma irradiation was performed

While unirradiated antibiotics did not exhibit any EPR signal, samples irradiated at room temperature showed complex EPR spectra (Fig. 2). The asymmetry of the vancomycin EPR spectra indicates their complex structure. According to the theory of EPR spectra shape, superposition of two different EPR lines can cause asymmetry of the resultant spectrum (Kęcki 1999; Stankowski and Hilczer 2005).

EPR spectra of a complex shape are observed for many irradiated medical substances (Basly et al. 1997, 1998c; Gibella et al. 2000). The reason for a complex shape of EPR spectra is the presence of several groups of free radicals (Basly et al. 1997, 1998c; Gibella et al. 2000, Stankowski and Hilczer 2005) in a sample, each of which results in a characteristic EPR line. EPR lines of particular groups of free radicals that contribute to an experimentally measured spectrum may be different in terms of amplitude, line width, and the value of the magnetic field resonant induction (Kęcki 1999, Stankowski and Hilczer 2005). Additionally, EPR lines of different types of free radicals are usually close to each other since their values of the magnetic field resonant induction are not much. Thus, the corresponding EPR lines overlap and it is difficult to identify them in the actually measured sum spectrum. Nevertheless, the measured spectra suggest the presence of more than one type of free radicals in the penicillin-derived antibiotics: piperacillin, ampicillin, and crystalline penicillin irradiated in the present study.

**Fig. 3:**
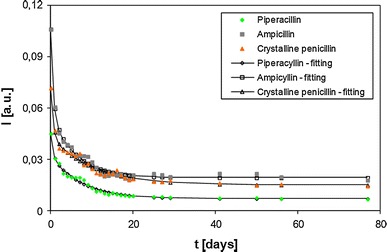
Time influence of sample storage on integral intensities (*I*) of EPR lines after gamma irradiation for piperacillin, ampicillin, and crystalline penicillin

**Fig. 4:**
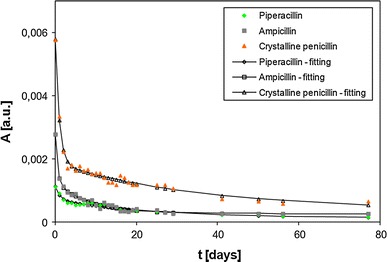
Time influence of sample storage on amplitudes (*A*) of EPR lines after gamma irradiation for piperacillin, ampicillin, and crystalline penicillin

Numerical analysis of the time dependence of the integral intensity (Fig. 3) and amplitude (Fig. 4) of the measured EPR lines demonstrated good agreement between the experimental points and the properly fitted two-exponential functions, which may also be a confirmation of the presence of two types of free radicals in the samples.

The observed decrease in free radical concentration as a function of storage time can be explained by the interaction of the free radicals with oxygen molecules O_2_ (Wilczyński et al. 2012). More specifically, this decrease may be due to the formation of quasi-chemical complexes between the free radicals of the sample and the paramagnetic molecules of oxygen O_2_. Pairing the spins of a free radical and an oxygen molecule causes the observed decrease in the intensity of EPR lines. Note that formation of quasi-chemical complexes between oxygen and a paramagnetic sample has been described in the literature (Pryor 1976). Presumably, the rapid decrease in the amount of free radicals in the first phase of samples storage corresponds to the interaction of oxygen with paramagnetic centers on the surface of the sample, where the oxygen supply is the best.

**Fig. 5:**
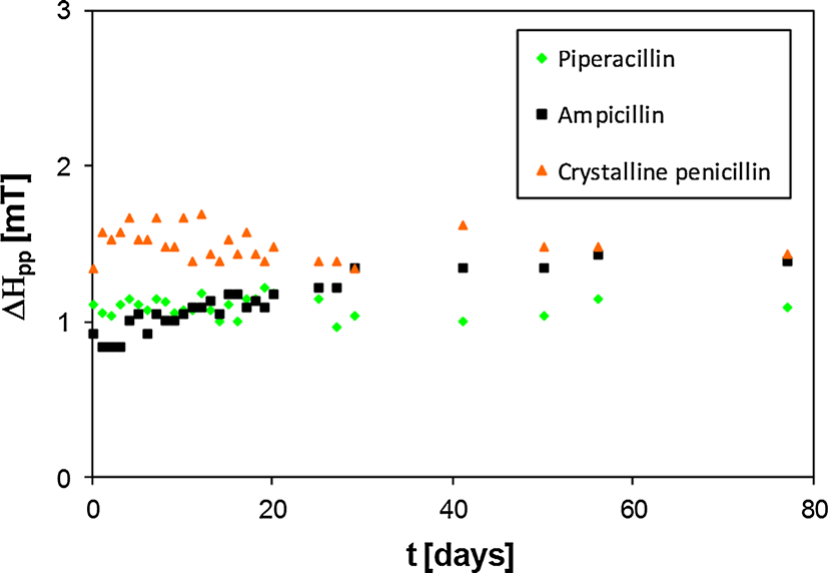
Time influence of sample storage on line widths (Δ*H*_pp_) of EPR lines after gamma irradiation for piperacillin, ampicillin, and crystalline penicillin

A substantial number of free radicals were present in the irradiated antibiotics (~10^17^ spin/g) (Table 1). The large EPR line widths (Δ*H*_pp_ 0.93–1.35 mT) suggest the distances between free radicals in the samples to be small (Table 1). The largest line widths were obtained for crystalline penicillin (Table 1). From the average g-factor values, the type of free radicals that are present in the samples can be determined. The obtained g-factor values indicate that in every irradiated antibiotic sample, the unpaired electrons were located on oxygen atoms (g = 2.0048–2.0062); however, this was not the case for crystalline penicillin where the unpaired electrons were located on sulfur atoms (g = 2.0083; Table 1). The g-values did not vary with storage time of the samples. In contrast, the concentrations of free radicals and EPR spectra parameters did change in all samples as a function of storage time. The changes in integral intensity (*I*), amplitude (*A*), and line widths (Δ*H*_pp_) of EPR lines as a function of storage time are shown in Figs. 3, 4, and 5, and the corresponding fit parameters obtained using a two-exponential function (Eqs. 1b and 2b) are given in Table 2.

**Table 1:** Free radicals concentrations (*N*) and their EPR spectra parameters: integral intensity (*I*), amplitude (*A*), line width (Δ*H*_pp_), and g-factor. The presented data pertain to EPR spectra recorded at 2 mW on the day of sample irradiation

Gamma-irradiated antibiotic	*N* × 10^17^ (spin/g) (10^17^)	*I* (a.u.)	*A* × 10^2^ (a.u.)	Δ*H*_pp_ (mT)	g-factor
Piperacillin	3.1 ± 0.1	0.3 ± 0.05	0.1 ± 0.05	1.11 ± 0.02	2.0062 ± 0.0002
Ampicillin	5.2 ± 0.1	0.5 ± 0.05	0.3 ± 0.05	0.93 ± 0.02	2.0048 ± 0.0002
Crystalline penicillin	2.0 ± 0.1	0.2 ± 0.05	0.6 ± 0.05	1.35 ± 0.02	2.0083 ± 0.0002

**Table 2:** Comparison of the parameters of the two-exponential functions *I*(*t*) and *A*(*t*) used, and *δ* error, for all investigated gamma-irradiated antibiotics

Gamma-irradiated antibiotic	*K*_0(*A*)_	*K*_0(*I*)_	*K*_1(*A*)_	*K*_1(*I*)_	*K*_2(*A*)_	*K*_2(*I*)_	*τ*_1(*A*)_	*τ*_1(*I*)_	*τ*_2(*A*)_	*τ*_2(*I*)_	*δ*_(*A*)_	*δ*_(*I*)_
Piperacillin	0.0001	0.01	0.0004	0.01	0.0006	0.02	1.04	0.51	24.97	7.48	0.00004	0.001
Ampicillin	0.0003	0.02	0.0015	0.05	0.0010	0.04	0.54	0.59	8.67	6.08	0.00004	0.002
Crystalline penicillin	0.0004	0.02	0.0039	0.03	0.0015	0.03	0.97	0.60	34.51	10.13	0.00009	0.001

**Fig. 6:**
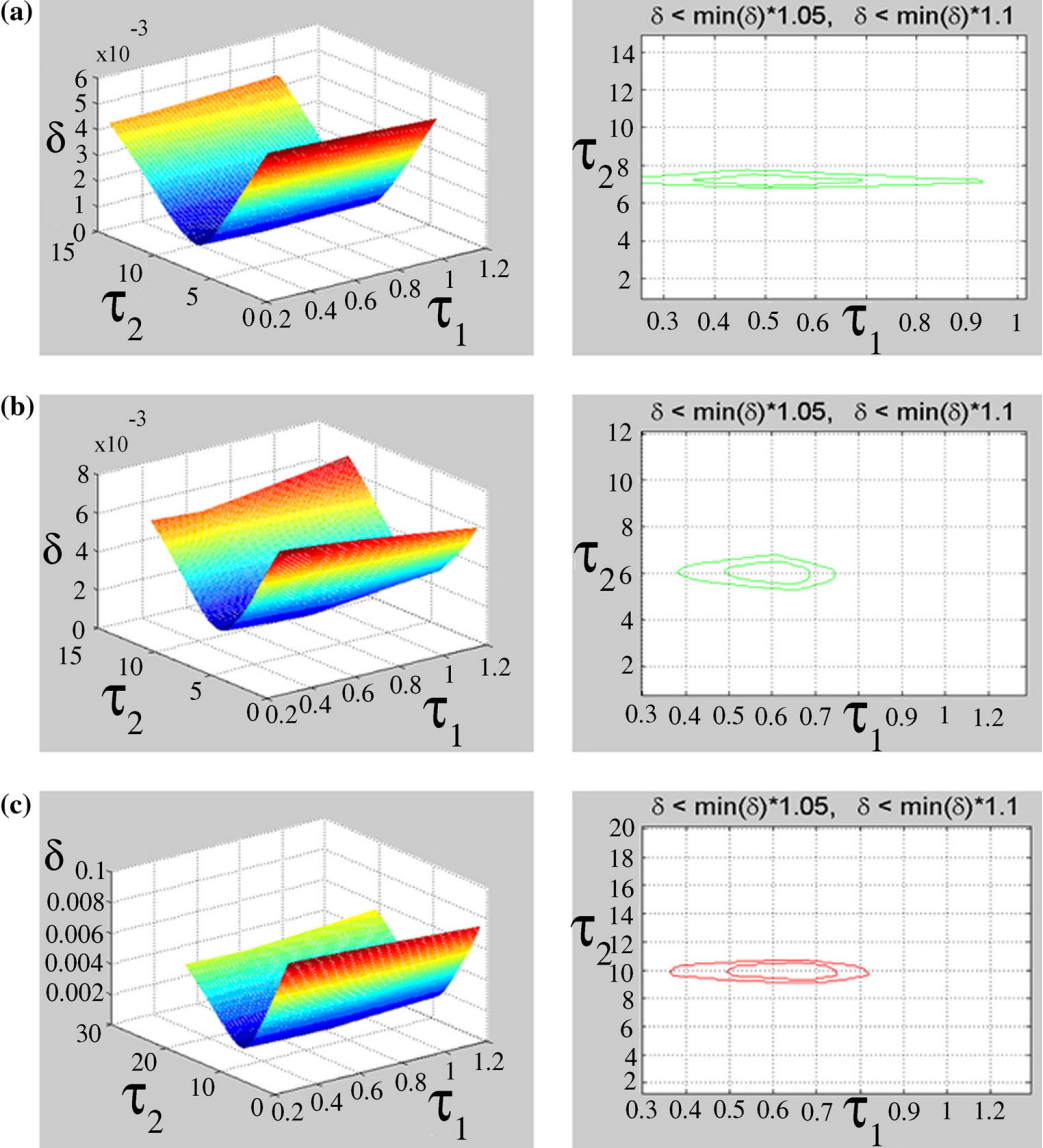
Dependence of the error *δ* after fitting the function given in Eq. 1b to the experimental data, on *τ*_1(*I*)_ and *τ*_2(*I*)_ (given in days) (*left figure panels*), and range of acceptable variation of *τ*_1(*I*)_ and *τ*_2(*I*)_ (in days), for piperacillin (**a**), ampicillin (**b**), and crystalline penicillin (**c**), respectively

**Fig. 7:**
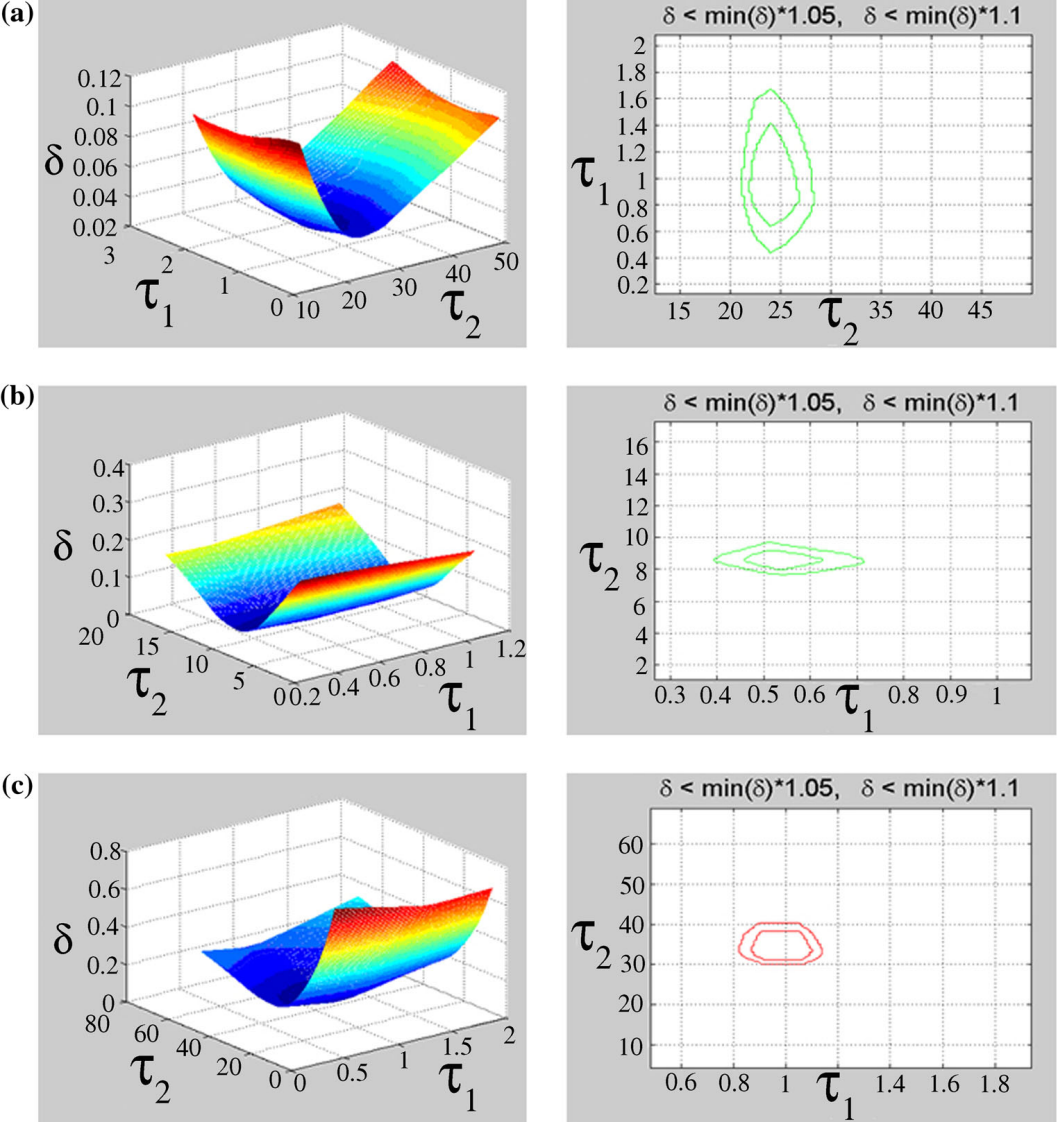
Dependence of the error *δ* after fitting the function given in Eq. 2b to the experimental data, on *τ*_1(*A*)_ and *τ*_2(*A*)_ (given in days) (*left figure panels*), and range of acceptable variation of *τ*_1(*A*)_ and *τ*_2(*A*)_ (in days), for piperacillin (**a**), ampicillin (**b**), and crystalline penicillin (**c**), respectively

The examined sensitivity of the *τ*_(*I*)_ parameters to the error defined as *δ* < min (*δ*) · 1.05 and δ < min (δ) · 1.10 indicates a relatively high sensitivity of the time constant *τ*_2(*I*)_ for all three gamma-irradiated samples of penicillin-derived antibiotics (piperacillin, ampicillin, and crystalline penicillin (Figs. 6 and 7)).

The change in the amplitude with storage time shows that in the first phase of samples storage (up to approximately 7 days), a rather fast decline of paramagnetic centers took place in ampicillin (*τ*_1(*A*)_ = 0.54 days), while a slightly slower decline was documented in piperacillin (*τ*_1(*A*)_ = 1.04 days) and crystalline penicillin (*τ*_1(*A*)_ = 0.97 days) (Table 2).

The time constants *τ*_2(*A*)_ show that the dynamics of the decrease in amplitude in the second phase of samples storage was similar to that in the first phase: again a faster decline was observed for ampicillin and a slower decline for piperacillin and crystalline penicillin (Table 2).

The examined sensitivity of the *τ*_(*A*)_ parameters to the *δ* error indicates a relatively high sensitivity of the time constant *τ*_2(*A*)_ for all examined samples (Table 2; Fig. 6). The range of acceptable variation in the parameters *τ*_1(*I*)_ and *τ*_2(*I*)_ with respect to the error δ is relatively narrow for gamma-irradiated crystalline penicillin (Table 2; Fig. 7). In contrast, samples of piperacillin and ampicillin show a greater sensitivity in terms of parameter *τ*_2(*I*)_ (Table 2; Fig 7).

Storage time did not affect the line widths of piperacillin and crystalline penicillin samples, whereas there was a small increase with time for ampicillin (Fig. 5).

Effects of gamma radiation on the color of the sample.

Gamma irradiation at 25 kGy caused discoloration, and the color of the samples changed from white to off-white. In contrast, there was no change in the color of the samples during their storage. After dissolving the samples in water, the re-dried antibiotics underwent slight color change, but did not return to the original color.

A higher concentration of radicals generated at the same absorbed dose of gamma radiation indicates a higher radiation sensitivity. In this context, however, the efficiency of microcrystalline matrices of trapping paramagnetic centers must also be considered. The present results suggest that dissolving the gamma-sterilized drug in water results in a rapid disappearance of free radicals. This may be due to the release of the paramagnetic centers from the crystal lattice of the drug and their recombination.

## Conclusions

From the pharmacological point of view, the biochemical properties of intermediates generated in drugs following gamma sterilization are important if they are formed in a significant number and therefore reduce the therapeutic properties of the pharmaceuticals. Free radicals formed in sterilized drugs may have an effect not only on the pharmacological activity of the drug (Polat and Korkmaz 2003), but also on its pharmacokinetic properties (Gibella et al. 2000), which is often neglected in the scientific literature.

Unfortunately, it is not possible to predict the influence of ionizing radiation on the stability of radicals even if the considered compounds are similar, because even minor changes in molecular structure may have a significant influence on processes observed by EPR spectroscopy (Polat and Korkmaz 2003). As expected, even though they belong to the same group of antibiotics, the sterilized substances investigated in the present study show very different properties under the influence of gamma radiation.

EPR spectra of the all analyzed irradiated antibiotics were complex in shape, demonstrating the existence of more than just one type of paramagnetic center. It has been proven here that the concentration of free radicals in the irradiated antibiotics decreases during storage (Figs. 3, 4). This decrease in the concentrations of free radicals is most pronounced shortly after irradiation and can be divided into two phases: (a) fast recombination (coefficient *τ*_1(*A*),(*I*)_) and (b) slow recombination (coefficient *τ*_2(*A*),(*I*)_). Phase 1 corresponds to surface recombination, while phase 2 to solid diffusion. In the case of a large number of paramagnetic centers and a short distance between them (during the first phase of storage), the possibility of a fast recombination of paramagnetic centers should also be considered.

It is assumed that the interaction between free radicals and oxygen molecules (O_2_), i.e., the pairing of free radical spins and oxygen molecules, is responsible for the decrease in free radicals concentration with time after irradiation (Wilczyński et al. 2012). The creation of quasi-chemical complexes between oxygen and radicals has already been described in the literature (Najder-Kozdrowska et al. 2010; Pilawa et al. 2002; Pilawa et al. 2003). In the present study, investigations using the EPR method have shown that free radicals in gamma-irradiated antibiotics are located in close proximity to each other. The values of EPR line widths for all tested antibiotics (0.93–1.35 mT) proved to be substantial (Table 1).

In general, wide EPR lines exist in the case of strong dipole spin–spin interactions and are characteristic for short distances of non-paired electrons of free radicals in the sample. The widening of EPR lines due to dipole interactions is caused by the statistical decay of the magnetic field in a sample (Pilawa et al. 2002).

Gamma radiation caused the formation of free radicals in a concentration of about 10^17^ spin/g, in the investigated antibiotics, and the largest concentration was found in gamma-irradiated ampicillin.

Important parameters to describe the behavior of free radicals in the samples are, among others, the time constants *τ*_1(*A*),(*I*)_ and *τ*_2(*A*),(*I*)_ and the constants *K*_0(*A*),(*I*)_, *K*_1(*A*),(*I*)_ and *K*_2(*A*),(*I*)_ of the considered exponential functions (Eqs. 1b and 2b). The time constants *τ*_1(*A*),(*I*)_, *τ*_2(*A*),(*I*)_ and the coefficients *K*_1(*A*),(*I*)_ and *K*_2(*A*),(*I*)_ quantify the decrease in the concentration of free radicals in an irradiated drug sample with time. The higher the *τ*_1(*A*),(*I*)_, *τ*_2(*A*),(*I*)_ parameters, the slower the decay of free radicals. The constant *K*_0(*A*),(*I*)_ indicates the concentration of free radicals in an irradiated drug sample after a long storage time. In the case of piperacillin, ampicillin, and crystalline penicillin, the constant *K*_0(*I*)_ was 0.01–0.02 for the integral intensity *I*(*t*), which indicates that during the sample storage time (76 days), the number of free radicals in the samples decreased to almost zero. At the same time, relatively low values of *τ*_1(*A*),(*I*)_ indicate a relatively rapid decrease in the amount of free radicals in the first phase of sample storage, while the higher values of *τ*_2(*A*),(*I*)_ indicate a slower decrease in the amount of free radicals during the second phase of samples storage. It can be also assumed that the exponent of the exponential function determines the number of types of free radicals in the samples. For the investigated samples (piperacillin, ampicillin, and crystalline penicillin), it is concluded that there are two types of paramagnetic centers, which is also confirmed by the complex nature of the spectrum.
